# A bibliometric analysis of the study of health equity of migrants

**DOI:** 10.1097/MD.0000000000044207

**Published:** 2025-09-05

**Authors:** Rui Min, Jiaxin Liu, Jiahui Tian, Fen Zhang, Jie Xiao

**Affiliations:** a School of Public Health, Tongji Medical College, Huazhong University of Science and Technology, Wuhan, China; b Hubei Provincial Center for Disease Control and Prevention, Wuhan, China; c The Second People’s Hospital of Yichang, Yichang, China.

**Keywords:** bibliometric analysis, health equity, migrants, research trend

## Abstract

**Background:**

Research on migrants has grown significantly over the past 20 years. However, systematic reviews and summaries of the health equity of migrants are lacking.

**Objective:**

This bibliometric analysis aims to reveal the knowledge structure, cooperation networks, and research frontiers in immigrant health equity for the first time, providing a framework and guidance for future studies.

**Methods:**

Publications related to health equity of migrants from 1993 to 2024 were collected from Scopus. The publications collected were restricted to certain categories (articles and reviews). VOSviewer and Citaspace were used to analyze the country/region, institution, research topic, keyword co-occurrence, and highly cited papers.

**Results:**

Four hundred nine articles were included in this study. It was found that the field entered a period of rapid growth after 2013, with an average of 29.67 articles per year, and the research enthusiasm has continued to rise. Articles originated from 63 countries/regions and 160 institutions. The study found that in terms of international cooperation and output, the United States (US) (55.74%), Canada, the United Kingdom, and China are the main research forces, with the University of California, San Francisco ranking first in the number of published papers; the US–United Kingdom, US–Canada, and US–China have formed the strongest cooperation networks. This study also reveals its interdisciplinary research characteristics, covering multiple fields such as medicine (75.31%) and social sciences (23.72%), and forms 5 core research clusters: healthcare services and social determinants: focusing on the impact of healthcare resource accessibility and socioeconomic status on health equity; demographic differences: paying attention to health inequalities caused by factors such as age, gender, and race; family income and Asian health: revealing for the first time the unique impact of family income on the health of Asian immigrants. Highly cited literatures highlight political factors (such as policy discrimination), group inequalities (such as the “Latino health paradox”), and methodological differences as academic focus, providing a theoretical basis for policy-making.

**Conclusions:**

The study confirms international and interdisciplinary trends. Future research should deepen multidisciplinary collaboration, explore group difference mechanisms, evaluate policies, strengthen international cooperation, use diverse methods, and support global immigrant health policy optimization.

## 1. Introduction

According to the International Organization for Migration, “migration” is “the movement of a person or group of persons across an international border or within the territory of a state.”^[1,2]^ Migration of the population can be subdivided into internal migration and international migration based on whether individuals cross national borders.^[2]^ In terms of internal migration, the largest internal migration is now occurring in middle-income countries, particularly China and India.^[3]^ Globally, international migration has become more and more prominent in recent years, with an estimated 281 million people in 2020, accounting for 3.6% of the global population. Among them, the United States (US) and Germany are the main destination countries of immigrants.^[4]^ The majority of “international migrants” are “migrant workers” engaged in paid activities, representing 62% of the total number of international migrants.^[5]^ In addition, international migration also includes people forced to be displaced by conflict, persecution, and other reasons, such as refugees and internally displaced persons.

Migrants and asylum seekers are at a higher risk of contracting diseases due to poor living conditions and large populations, and are often constrained by the host country’s political system, resulting in limited access to healthcare services.^[6,7]^ The interweaving of national and international migration makes the health of migrants more complex and dynamic. At present, health problems of migrants have attracted increasing attention from the international community.

The relationship between migration and health is complicated, and its effects are highly variable among and within migrant groups.^[8]^ The risk and pathogenic factors of health not only involve individual factors such as genetics and nutrition, but also are closely related to the inequality mechanism in social structure.^[9]^ For migrants, the issue of health equity is particularly salient. Migration is not only the flow of geographical location but also the reconstruction of social relations and cultural cognition. After migration, migrants’ health behaviors, especially risk behaviors, become dynamically sensitive to social relationships in both their country of origin and destination, thereby increasing their risk of health problems.^[9,10]^

Faced with health disparities between ordinary citizens and migrants, the World Health Organization (WHO) has proposed the slogan “leaving no one behind” and urges countries to put the health of migrants on their agenda and take concrete steps to improve their health. In addition, on WHO’s 75th anniversary of the WHO, Director-General Tedros Adhanom Ghebreyesus called for global cooperation to achieve health equity, including that of migrants.^[11]^ Health equity means that everyone has the fair and just opportunity to be as healthy as possible.^[12,13]^ Achieving health equity for migrants, it is imperative to address the various obstacles they face, such as socioeconomic disadvantage and discriminatory practices, including powerlessness and lack of access to good, equitably paid jobs, quality education, housing, health care, and a safe environment, need to be removed.^[13]^ In addition, migrants and their families often lack the legal status to access basic health care, and when available, they often avoid seeking care due to language, cultural, financial barriers, and fear of expulsion, xenophobic, and discriminatory attitudes.^[14]^

At WHO’s urging, international organizations and receiving nations have responded positively, and the health status of migrants has improved. However, the issue of health equity among migrants remains persistent, and research on health equity of migrants has increasingly become the focus of migration research. This study maps the knowledge structure, research trends, and popular topics in the field of health equity for migrants. To our knowledge, this is the first exhaustive bibliometric analysis of migrant health equity. For the first time, this study conducts a comprehensive analysis of 409 literatures in the field of immigrant health equity from 1993 to 2024 using bibliometric methods, filling 3 major research gaps: firstly, with the help of VOSviewer and CiteSpace tools, it reveals for the first time the national cooperation network, institutional output pattern and keyword co-occurrence clusters in this field, providing researchers with a “field map” to visualize the knowledge structure. In addition, although this study covers multiple disciplines such as medicine, social sciences, and psychology, existing studies lack a systematic review of interdisciplinary intersections. This study identifies a 3-dimensional research framework of “healthcare services–social determinants–demographic characteristics” through keyword clustering, which provides a basis for breaking disciplinary barriers. Meanwhile, the discovery of the 5 research clusters reveals the mechanisms of group differences that have been neglected in traditional studies, offering a forward-looking perspective for policy-making.

**Figure 1.**
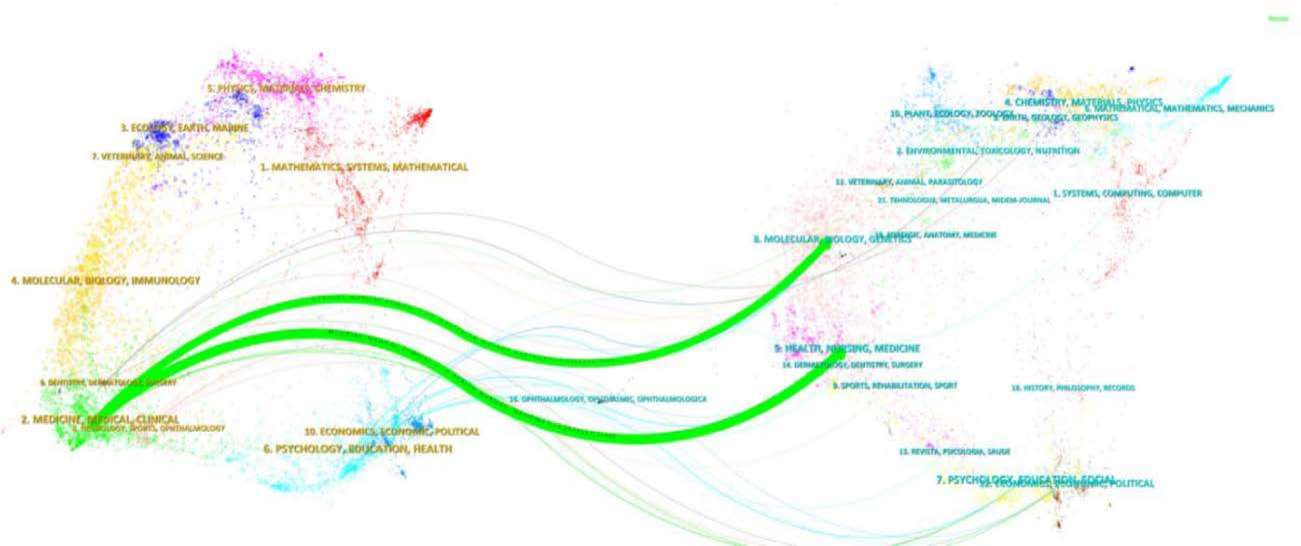
Double graph superposition of citations of articles. The left side shows the citing journals, and the right side shows the cited journals. Curved paths between them represent citation relationships, illustrating the interdisciplinary citation connections in the field.

The use of bibliometric analysis to explore immigrant health equity can more systematically reveal the multi-dimensional complexity and comprehensiveness of the research scope in this field. Compared with traditional narrative reviews, it can quantify the degree of interdisciplinary integration. For example, Figure 1 can intuitively present the citation links between medical journals and social science journals, confirming the actual existence of disciplinary collaboration and potential gaps. In addition, it can better reflect the dynamic growth trend and capture long-term cycles. The co-citation analysis of highly cited literatures, rather than subjective induction, further enhances the objectivity of this study.^[15]^

This study, through bibliometric analysis, achieves a leap from “fragmented research” to “systematic cognition”: it not only presents a “research map” in the field of immigrant health equity, but also identifies key gaps through quantitative evidence: such as insufficient research in emerging market countries (China accounts for only 5.62%) and weak analysis from an economic perspective (with only 6 economics-related literatures), providing a “problem-oriented” improvement path for future research. Compared with traditional qualitative reviews, this big data-based review method is more adaptable to the dynamics and complexity of immigrant health issues, and provides a scientific basis for the optimization of global immigrant health policies.

## 2. Methods

### 2.1. Data sources and search strategy

The study data were obtained from the Scopus database, which covers a broad spectrum of disciplines related to migrant health equity, including medicine and the social sciences. This database was chosen because of its global recognition in terms of authority, credibility, comprehensiveness, applicability, and popularity in bibliometric analysis.^[16–19]^

Given that the equitable research of immigration health has significant interdisciplinary attributes and needs to meet the needs of long-cycle data backtracking and complex network analysis, the Scopus database has more advantages in terms of interdisciplinary and visualization. Its interdisciplinary coverage can effectively integrate literature in nonmedical fields such as social sciences and avoid the disciplinary limitations of PubMed. Compared with Web of Science, Scopus provides more complete long-cycle data and more comprehensive early literature inclusion. In terms of the adaptability of analytical tools, Scopus is highly compatible with VOSviewer and CiteSpace, and supports multi-dimensional indicator analysis such as “Total Link Strength.” In addition, Scopus’s institutional matching algorithm and national classification are more accurate than PubMed’s MeSH terminology restrictions. These characteristics make it an ideal choice for interdisciplinary, long-cycle immigration health equity research, especially suitable for complex network analysis and trend visualization research.

Taking Scopus as a representative case, the search strategy was developed on June 30, 2023, and updated on December, 2024. Two search strategies have been developed for immigrants and health equity. The search strategy was as follows.

(TITLE-ABS-KEY (“international” OR “overseas” OR “cross-border” OR “non-citizen*” OR “non-national*” OR foreign* OR transnational* OR expatriate* OR alien* OR transient*) OR TITLE-ABS-KEY (migrant* OR migrat*) OR TITLE-ABS-KEY (refugee* OR “non-asylum”) OR TITLE-ABS-KEY (stateless PRE/2 person* OR population* OR people) OR TITLE-ABS-KEY (mobile OR mobility OR movement* OR displace* OR travel*)) AND (TITLE (“health* disparit*” OR “health care disparit*” OR “disparit* of health*” OR “disparit* of health care”) OR TITLE (“health* equalit*” OR “health care equalit*” OR “equalit* of health*” OR “equalit* of health care” OR “health* equit*” OR “health care equit*” OR “equit* of health*” OR “equit* of health care”) OR TITLE (“health* inequalit*” OR “health care inequalit*” OR “inequalit* of health*” OR “inequalit* of health care” OR “health* inequit*” OR “health care inequit*” OR “inequit* of health*” OR “inequit* of health care”)) (Table 1).

**Table 1:** Search strategy and number of retrieved publications in Scopus.

No.	Strategy	Search query (applied in Scopus)	*N*
1	Applied migrant, migration, and mobility search query in the publication title, publication source, and keywords	TITLE-ABS-KEY (“international” OR “overseas” OR “cross-border” OR “non-citizen*” OR “non-national*” OR foreign* OR transnational* OR expatriate* OR alien* OR transient*) OR TITLE-ABS-KEY (migrant* OR migrat*) OR TITLE-ABS-KEY (refugee* OR “non-asylum”) OR TITLE-ABS-KEY (stateless PRE/2 person* OR population* OR people) OR TITLE-ABS-KEY (mobile OR mobility OR movement* OR displace* OR travel*)	8,575,957
2	Applied health disparities search query in the publication title	TITLE (“health* disparit*” OR “health care disparit*” OR “disparit* of health*” OR “disparit* of health care”) OR TITLE (“health* equalit*” OR “health care equalit*” OR “equalit* of health*” OR “equalit* of health care” OR “health* equit*” OR “health care equit*” OR “equit* of health*” OR “equit* of health care”) OR TITLE (“health* inequalit*” OR “health care inequalit*” OR “inequalit* of health*” OR “inequalit* of health care” OR “health* inequit*” OR “health care inequit*” OR “inequit* of health*” OR “inequit* of health care”)	13,717
3	Combined strategies 1 and 2	1 AND 2	1418
4	Limited literature category, article	LIMIT-TO (Literature category, Article and Review)	1138
5	Excluded irrelevant publications identified by title and abstract screening (using Scopus saved list and MS Excel)		409

Search strategy was developed on June 30, 2023 and updated on December 2024.

MS Excel = Microsoft Excel, N = number of publications retrieved.

The language was limited to English, the document type was limited to articles and reviews, and the search time was 1993 to 2024.

A total of 1138 documents were retrieved from the Scopus database. After excluding irrelevant articles, 409 were retained for inclusion in the study. Table 1 presents the full search strategy. Independent and specific literature selection was conducted through a collaboration between the 2 authors.

**Figure 2.**
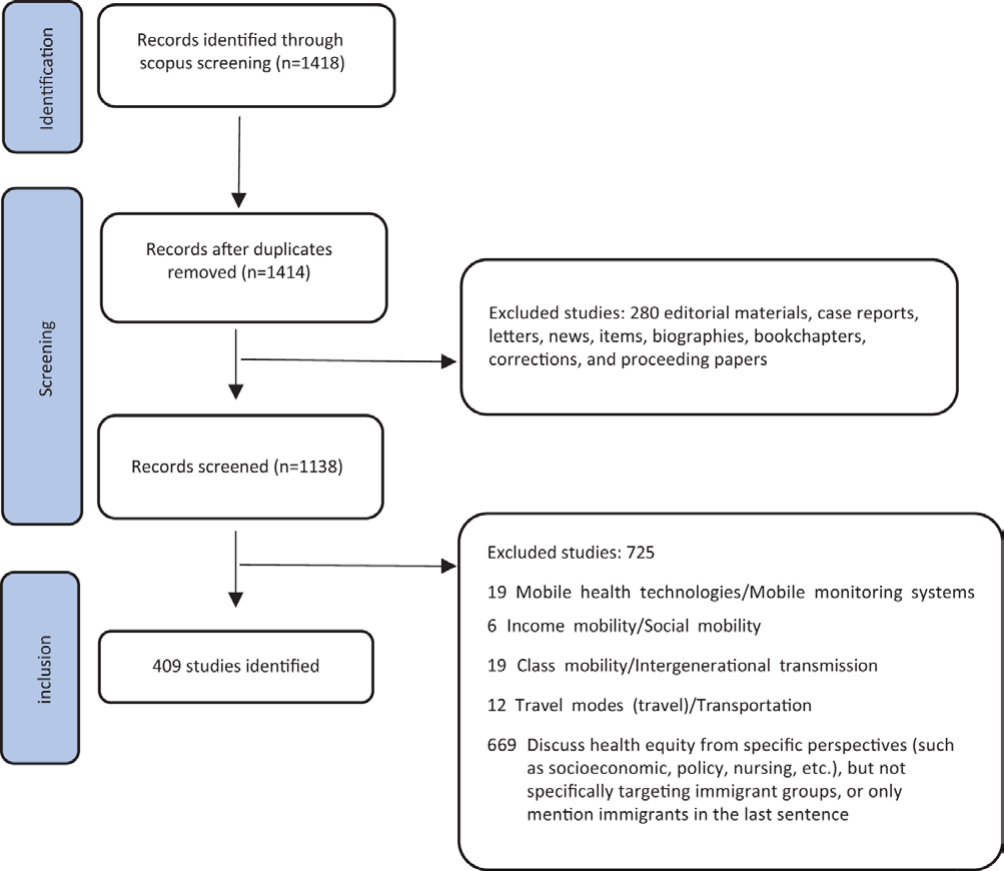
Bibliometric review flow diagram. Flow diagram depicting the step-by-step process of study identification, screening, and exclusion for a new bibliometric review, which was based solely on database and register searches. The key stages are as follows: identification: 1418 records were initially identified through Scopus screening. After duplicates removed: 1414 records remained following the elimination of duplicate entries. Screening: 1138 records underwent eligibility screening. Exclusions: a total of 725 studies were excluded during screening. A total of 409 studies were finally retained. The diagram illustrates the progressive reduction in the number of records throughout the review process.

Exclusion criteria: exclude duplicate articles (use Excel to filter unique DOIs and delete duplicates). Exclude non-article or non-review literatures, such as editorial materials, case reports, letters, etc. Exclude articles irrelevant to the themes of “health equity” and “immigrants,” such as those about mobile health technologies/mobile monitoring systems, income mobility/social mobility, etc. Exclude studies that do not focus on the target population of “immigrants.” The specific exclusion strategy is shown in the BIBILO flow diagram (Fig. 2).

### 2.2. Ethical approval and consent to participate

The study protocol and contents were approved by the Medical Ethics Committee of Tongji Medical College, Huazhong University of Science and Technology (ethics approval code S182).

### 2.3. Data extraction and analysis

Scopus and Microsoft Excel (Redmond) were used to analyze bibliometric information, including authors, publications, and journals. Scopus is commonly used as a reference database for bibliometric analysis. It has a built-in analysis function that can generate a list of leading publications, journals, authors, country–author affiliations, institutions or organizations, aggregates of publication types, and subject areas.^[20–22]^ Microsoft Excel was used to produce the counts and percentages of publications by theme, subtheme, migrant topic, and country topic/coverage.

The total number of publications was plotted using Origin Pro 2024 (OriginLab Corporation, Northampton). The VOSviewer (version 1.6.19), which was created by Van Eck and features a robust bibliometric mapping function, was used to map the relationships between countries, institutions, journals, and author collaborations, which was created by Van Eck and features a robust bibliometric mapping function.^[23]^ Additionally, the VOSviewer can group and synchronously colorize keywords with high co-occurrence frequencies into several clusters.^[23]^ Research trends and hotspots were determined using a co-occurrence analysis. As the basis for our investigation, “author keywords” were selected.

## 3. Results

### 3.1. Analysis of overall trends in publications

**Figure 3.**
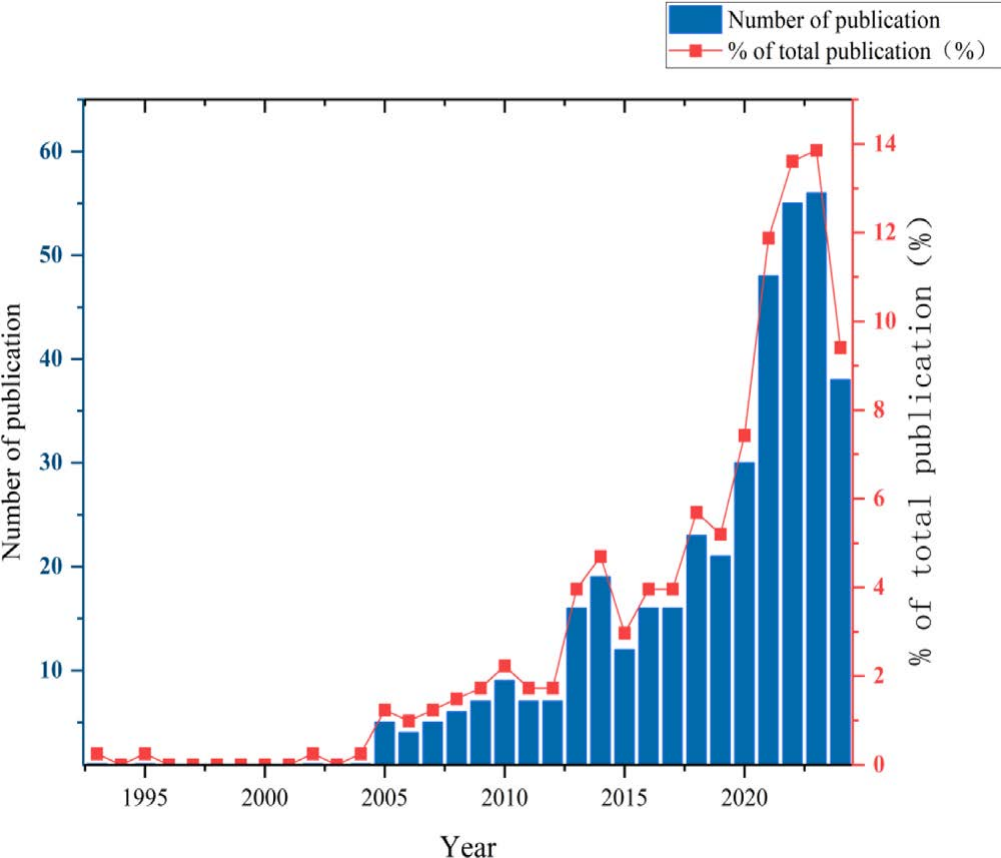
Total publications from 1993 to 2024. The trends in the number of publications on migrant health equity over the 31-year period, divided into 3 stages: gestation period (1993–2004), slow growth period (2005–2012), and rapid growth period (2013–2024).

Figure 3 depicts the trends in publications on migration health equity. Our search yielded 409 articles covering 1993 to 2024. From when the first article was published in 1993 until 2024, the number of articles published continuously increased, with an obvious overall growth trend. In terms of average annual publications, the trend can be divided into 3 stages: a gestation period (1993–2004) with an average of 0.33 publications per year, a slow growth period (2005–2012) averaging 6.25 publications annually, and a rapid growth period since 2013, averaging 29.67 publications per year (as of December 2024).

### 3.2. Analysis of country/region and institutions distribution

Regarding distribution by country/region and institution, publications spanned 63 countries/regions and 160 institutions. Table 2 lists the countries with the highest production, with the US being the most active country in the field, accounting for 55.74% of the 409 papers analyzed. Canada, the United Kingdom (UK), and China followed closely with percentages of 8.56%, 8.31%, and 5.62%, respectively. The University of California, San Francisco, was the most productive institution, publishing 31 papers, accounting for 7.58% of all the publications (Table 3).

**Table 2:** List of most productive* countries by corresponding authors on health equity of migrants.

Rank	Country	*N*	%
1	United States	228	55.74
2	Canada	35	8.56
3	United Kingdom	34	8.31
4	China	23	5.62
5	Australia	18	4.40
6	Italy	16	3.91

N = total number of publications (as of December 2024).

* Countries with 15 or more publications in Scopus.

**Table 3:** List of most productive* institutions.

Rank	Institutions	*N*	%
1	University of California, San Francisco	31	7.58
2	The University of British Columbia	10	2.44
3	University of California, Los Angeles	9	2.20
4	University of Minnesota Twin Cities	9	2.20
5	Johns Hopkins University	8	1.96
6	University of Michigan, Ann Arbor	8	1.96
7	Johns Hopkins University School of Medicine	7	1.71
8	Harvard University	7	1.71
9	University of Ottawa	7	1.71

N = total number of publications (as of December 2024).

* Institutions with 7 or more publications in Scopus.

**Figure 4.**
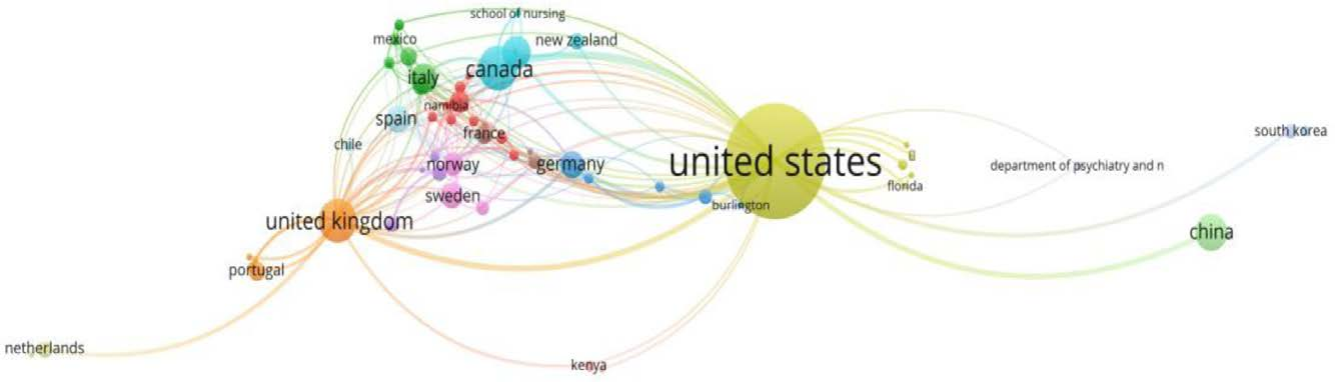
Countries/regions co-authorship network. The color represents different clusters of collaborating countries/regions. Nodes denote countries/regions, with larger nodes indicating a higher number of published articles. Links between nodes represent collaborative partnerships between the respective countries/regions.

Using VOSviewer, we drew a network visualization map that depicted the largest sets of international research collaborations among countries active in the field of health equity for migrants (Fig. 4). The map indicates that the strongest international research collaborations were between the US and the UK, followed closely by those between the US and Canada and then between the US and China.

### 3.3. Analysis of journal distribution

The 409 articles cited in this study have been published in 160 journals. Table 4 lists the most productive journals, based on the number of publications. Social Science and Medicine ranked first (17/409, 4.16%). The dual map shows 2 main reference paths. The left side shows the research frontier, with articles concentrated in journals in the medical, neurological, and clinical fields, while the right side shows the cited region, with articles primarily published in journals in the molecular, biological, genetic, health, nursing, and medical fields (Fig. 1).

**Table 4:** List of most productive* journal.

Rank	Productive journal	*N*	%	IF	Q
1	Social Science and Medicine	17	4.16	4.9	Q1
2	International Journal of Environmental Research and Public Health	16	3.91	4.614	Q1
3	International Journal for Equity in Health	14	3.42	4.5	Q1
4	Journal of Immigrant and Minority Health	11	2.69	2	Q3
5	Frontiers in Public Health	10	2.44	3	Q2
6	BMC Public Health	9	2.20	4.5	Q2
7	PLOS One	7	1.71	2.9	Q1
8	Revista Panamericana de Salud Publica/Pan American Journal of Public Health	6	1.47	2	Q3
9	American Journal of Industrial Medicine	5	1.22	2.7	Q2
10	BMJ Open	5	1.22	2.4	Q1
11	European Journal of Public Health	5	1.22	4.4	Q2
12	SSM Population Health	5	1.22	4.7	Q2

IF = impact factor, N = total number of publications (as of December 2024), Q = quartile in category, represents the JCR (Journal Citation Reports) partition.

* Journal with 5 or more publications in Scopus.

### 3.4. Analysis of research topics

We investigated immigrant health equity research topics based on categories and themes. The top 11 publications are presented in Table 5. “Medicine” (308/409, 75.31%), “social sciences” (97/409, 23.72%), and “arts and humanities” (32/409, 7.82%) were the 3 most important research categories. In addition, other categories included “nursing”, “environmental science”, “psychology”, “biochemistry, genetics, and molecular biology”, “dentistry”, “health professions”, “multidisciplinary”, and “economics, econometrics, and finance.”

**Table 5:** List of most productive* research categories.

Rank	Scopus categories	*N*	%
1	Medicine	308	75.31
2	Social Sciences	97	23.72
3	Arts and Humanities	32	7.82
4	Nursing	29	7.09
5	Environmental Science	29	7.09
6	Psychology	20	4.89
7	Biochemistry, Genetics and Molecular Biology	10	2.44
8	Dentistry	10	2.44
9	Health Professions	9	2.20
10	Multidisciplinary	7	1.71
11	Economics, Econometrics and Finance	6	1.47

* Research categories with 5 or more publications in Scopus.

### 3.5. Analysis of co-occurrence of keywords

Table 6 shows the top 10 keywords according to their occurrence. The most frequently occurring keyword was “human,” which appeared 335 times, with a total link strength of 6040. In addition, “health status disparities”, “female”, “adult”, and “male” were also frequently used keywords that ranked highly in both occurrence frequency and connection strength.

**Table 6:** List of most productive* keywords of health equity of migrants.

Rank	Keywords	Occurrences	Total link strength
1	Human	335	6040
2	Health disparity	216	4148
3	Female	182	3793
4	Adult	159	3300
5	Male	159	3300
6	Migrant	108	2203
7	Unites states	106	1907
8	Migration	95	1811
9	Emigrants and immigrants	93	1835
11	Health equity	92	1359

Keywords with ≥ 80 occurrences in Scopus.

**Figure 5.**
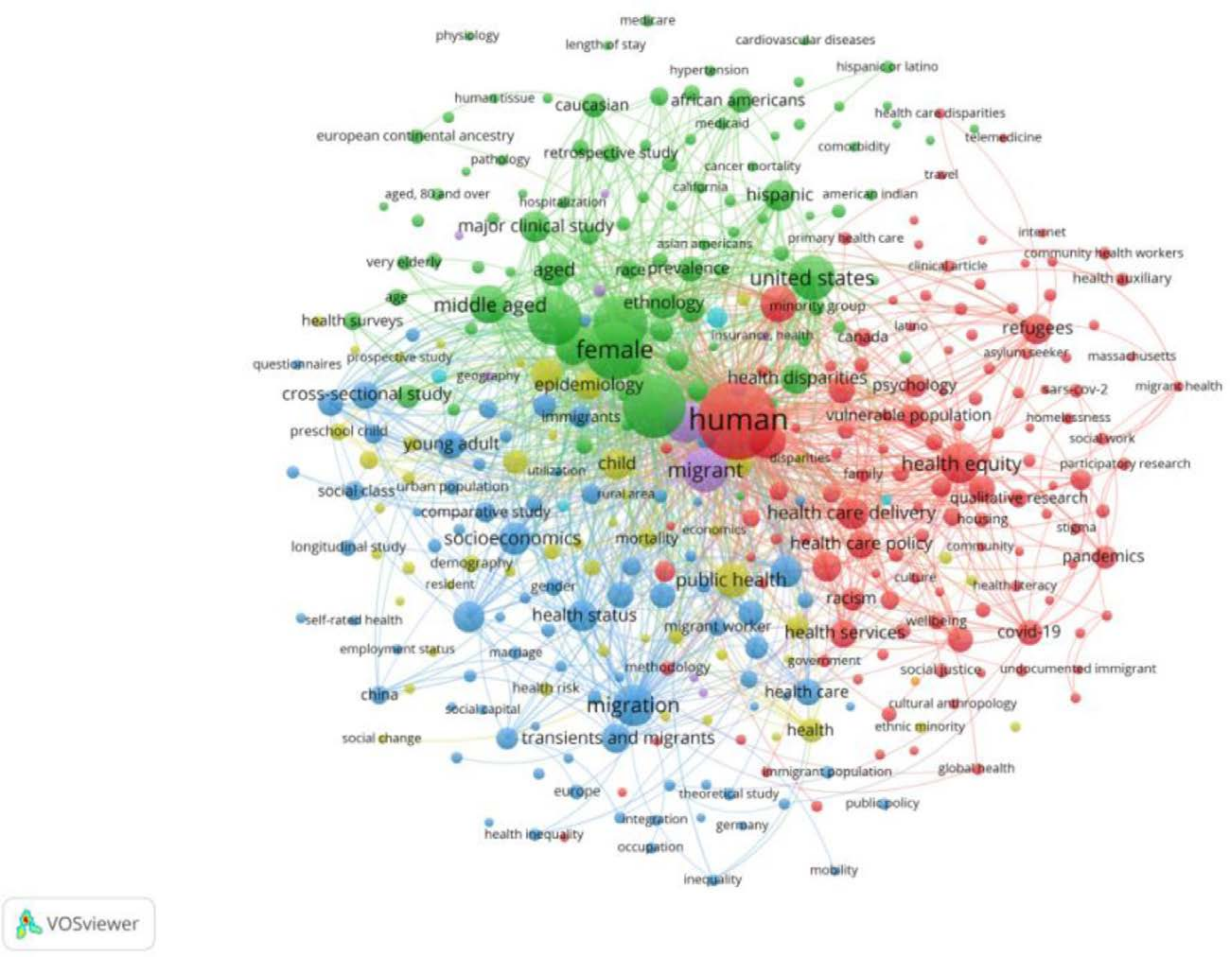
Keyword co-occurrence network. Colors represent different research clusters. Nodes denote keywords, with larger nodes indicating higher occurrence frequencies of the keywords. Links between nodes reflect co-occurrence relationships, highlighting the core research themes and their connections.

The 5 clusters are shown in Figure 5. The red cluster focuses on migration and healthcare factors, including keywords such as “human” (n = 335), “health equity” (n = 92), “healthcare disparities” (n = 216) and “health care delivery” (n = 59).

The core keywords in the blue cluster are “migration” (n = 95), “socioeconomics” (n = 58), “social determinants of health,” (n = 56), “social status” (n = 32), and “China” (n = 21). These keywords emphasize the social determinants of health for migrants.

The yellow cluster is largely related to the age of the migrants. The core keywords “child” (n = 53), “young adult” (n = 44) and “comparative study” (n = 25), and “new born.” These studies described health disparities by age, mainly by using comparative studies.

The green cluster is primarily linked to the gender and ethnicity of migrants. The core keywords “male” (n = 159), “female” (n = 182), “Unites states” (n = 106), and “Hispanic” (n = 54). These studies describe health disparities according to sex and race, particularly in the US.

The keywords of the purple cluster include “Asian Americans” (n = 32), “emigrants and immigrants” (n = 93), “household income” household income’(n = 6). The main research topic is the impact of household income on the health of Asian-Americans.

**Figure 6.**
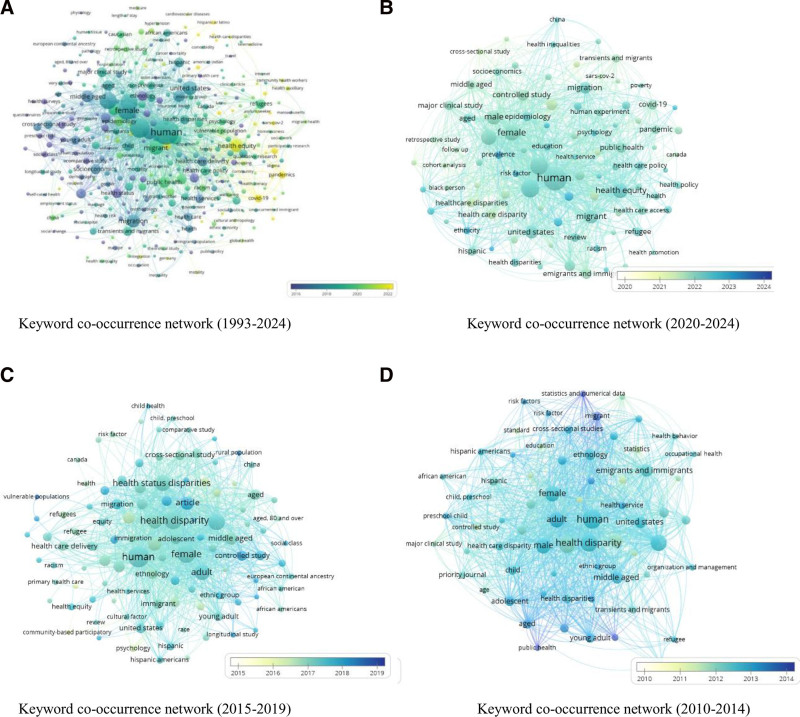
Keyword co-occurrence networks and overlay visualizations. Colors represent temporal trends of keyword usage. Nodes denote keywords, with larger nodes indicating higher occurrence frequencies. (A–D) The keyword networks for the periods 1993 to 2024, 2020 to 2024, 2015 to 2019, and 2010 to 2014, demonstrating the evolutionary dynamics of research hotspots over time.

To better elucidate the co-occurrence relationship between health equity of migration keywords and changing trends, 366 distinct keywords that appeared in 5 or more occurrences were identified using the VOSviewer after replacing or removing synonyms. Figures 6 shows the network and overlay visualizations of keyword co-occurrence. It shows the time-dependent evolution of the research trend.

Figure 6A presents the keyword co-occurrence network from 1993 to 2024, which covers the research theme associations in the field of migrant health equity over nearly 30 years. Nodes represent various keywords, with colors changing over time to intuitively reflect the activity of keywords in different periods; the size of nodes is positively correlated with the frequency of keyword occurrences, and the links between nodes indicate the co-occurrence relationships of keywords. Overall, this network not only shows the continuous influence of traditional research themes such as “healthcare accessibility” and “social determinants” but also reveals, through the temporal distribution and association changes of keywords, the evolution trend of global migrant health equity research from early focus on basic health issues to multi-dimensional and cross-regional comprehensive analysis.

In the early days (2010–2014), the network structure was simple, and the core nodes were concentrated on basic methods such as information retrieval and statistical analysis. Clusters were scattered and the connections were sparse, reflecting the singleness of the start-up period of the discipline (Figure 6D).

In the mid-term (2015–2019), research focused on social structural factors and health equity among specific populations. It centered on the impact of social stratification, ethnicity, and other factors on health disparities, with attention paid to groups such as children and ethnic minorities. Through community-based participatory research and other methods, the root causes were analyzed, providing micro-level empirical evidence for health equity policies (Fig. 6C).

Recently (2020–2024), driven by the coronavirus disease 2019 (COVID-19) pandemic, research shifted towards infectious disease prevention and control, public health emergency response, and the protection of vulnerable groups. The research context expanded to health equity in the context of the pandemic, with methods placing more emphasis on timeliness and adaptability, and the perspective becoming more global. It focused on health equity in emergency situations and macro-level intervention strategies (Fig. 6B).

In the evolution of research trends, health equity has remained the core thread. The early stage focused on analyzing root causes, while the later stage emphasized crisis response, with the research objects expanding from specific groups to a broader range of vulnerable groups. This change, driven by major public health events, not only revealed loopholes in the health equity framework but also promoted the emergence of practical tools such as the “Guidelines for Protecting Vulnerable Groups During the Pandemic.” It reflects the role of major social events in reshaping research hotspots and also reveals the lessons learned by health policies in responding to major public health emergencies.^[24]^

### 3.6. Analysis of highly cited articles

We also analyzed 11 high-impact articles cited more than 200 times; the specific information is shown in Table 7. These highly cited articles have not only aroused wide attention in the academic community, but have also revealed the key factors affecting the health equity of immigrants.

**Table 7:** List of most productive* cited articles.

Title	Authors	Citations	Journal	IF
Why cultural safety rather than cultural competency is required to achieve health equity: A literature review and recommended definition	Curtis E et al	759	International Journal for Equity in Health	4.5
The political origins of health inequity: Prospects for change	Ottersen OP et al	436	The Lancet	98.4
Racial, economic, and health inequality and COVID-19 infection in the United States	Abedi V et al	435	Journal of Racial and Ethnic Health Disparities	3.2
Narrative versus nonnarrative: The role of identification, transportation, and emotion in reducing health disparities	Murphy ST et al	404	Journal of Communication	6.1
Health disparities in the latino population	Vega WA et al	335	Epidemiologic Reviews	5.2
Work organization, job insecurity, and occupational health disparities	Landsbergis PA et al	294	American Journal of Industrial Medicine	2.7
Migration-related health inequalities: Showing the complex interactions between gender, social class and place of origin	Malmusi D et al	269	Social Science and Medicine	4.9
State-level immigration and immigrant-focused policies as drivers of Latino health disparities in the United States	Philbin MM et al	265	Social Science and Medicine	4.9
Understanding the micro and macro politics of health: Inequalities, intersectionality & institutions – A research agenda	Gkiouleka A et al	247	Social Science and Medicine	4.9
Prevalence of mental disorders and utilization of mental health services in two American Indian reservation populations: Mental health disparities in a national context	Beals J et al	259	American Journal of Psychiatry	14.7
Eviction, health inequity, and the spread of COVID-19: Housing policy as a primary pandemic mitigation strategy	Benfer EA et al	208	Journal of Urban Health	4.3

IF = impact factor.

* Articles with citation frequencies >200.

The studies presented in Table 7 can be divided into several main. Firstly, the highly cited article put forward the theory of “the political roots of health inequality” for the first time. Some studies have pointed out that political factors are important sources of health inequality among migrants. This study analyzes how globalization-related activities reshape health risks, noting conflicts between health sector efforts and global interest-pursuing actors exacerbating inequities. Political bias and discriminatory policies hinder migrants’ access to healthcare and social services, adversely affecting their health.^[25]^ Additionally, country-level policies significantly impact migrant health, with US immigration policies affecting Latinos through structural racism and restricted access to resources. Existing research often oversimplifies policy impacts, suggesting the need for multi-sectoral, multi-level, and multi-outcome approaches.^[26]^ Through the analysis of highly cited articles, this study has promoted the academic community to shift from a single perspective of “healthcare accessibility” to a multi-dimensional analysis of “political-social structure.”

Secondly, highly cited articles have revealed health inequalities among specific groups and put forward the “Latino health paradox” for the first time: Latin American immigrants face fewer fatal health issues than non-Latino white immigrants, but this advantage does not extend to their children. Socioeconomic heterogeneity among Latinos is notable, with mortality differences observed according to socioeconomic status (SES). The SES gap narrows with age, with low-SES Latinos benefiting the most. Low-income Latinos bring protective health behaviors to the US and are less likely to adopt potentially harmful new behaviors. It challenges the assumption that “immigrant health disadvantages inevitably intensify with social integration” and reveals the impact of intragroup heterogeneity on health equity. Subsequent studies have further found that undocumented Latino immigrants avoid seeking medical treatment due to fear of policy penalties, and their health advantages are actually the result of “detection bias.” This has promoted research to shift from “phenomenon description” to “mechanism deconstruction.”

The third focus area is research perspectives and methods on migrant health inequalities. First highly cited studies have quantified ethnic-immigrant disparities in pandemic-related infection and mortality, integrating structural determinants such as eviction and housing policies into the health equity framework and thereby establishing a new paradigm for addressing health inequities during global public health emergencies. Second, studies have contrasted narrative and nonnarrative approaches to reducing health inequalities, emphasizing the importance of emotion, identification, and substitution. Narrative formats impact knowledge, attitudes, and behaviors among diverse American groups, particularly Mexican Americans.^[27]^ It provides an interdisciplinary path for health education. On the other hand, an integrated framework combining intersectionality and institutional approaches is proposed to address the complexity of individual social positioning and institutional stratification in health. These studies advance the research on health inequality and support comprehensive health policy development.^[28]^

The significance of highly cited articles in the field of migrant health equity research stems from their role in reshaping the core directions of the research landscape through theoretical breakthroughs and methodological innovations. From the perspective of political factors, highly cited articles were the first to incorporate national policy discrimination and structural racism as core variables, promoting “immigrant policies and health equity” from a marginal topic to an independent research field and driving advancements in this area. In research on group differences, the “Latino health paradox,” supported by 335 citations, has systematically resolved academic controversies over “immigrant health advantages,” directly spawning subfields such as Asian immigrant health. Meanwhile, the impact of household income on health has emerged as a new focus, demonstrating the leading role of highly cited articles in shaping the research landscape. At the methodological level, highly cited articles have promoted the standardization of narrative research methods and intersectional analytical frameworks, facilitating the widespread application of narrative approaches in the field. However, there is a lag in certain areas: narrative methods have been slow to gain traction in Asian countries due to cultural differences, and the lack of highly cited articles from an economic perspective has created a theoretical gap relative to the actual impact of immigrants’ economic status on health equity. Overall, the citation frequency of highly cited articles is highly consistent with their theoretical importance in deconstructing political factors, explaining group paradoxes, etc. Nevertheless, there are timeliness deficiencies in cross-cultural methodological applications and economic mechanism analysis. Future efforts should focus on strengthening theoretical coverage of emerging groups, constructing dynamic policy evaluation models, and integrating big data with bibliometric tools, while continuing interdisciplinary approaches to fill research gaps.

Highly cited literature indicates that the core of health inequalities among immigrants lies in the intersecting effects of structural social determinants (housing discrimination, economic exclusion, legal status barriers) and regionally differentiated risks. It is thus suggested that health equity should be incorporated into the main thread of immigration policies, adopting a 3-in-one strategy of “legalization of legal status + economic safety net + culturally appropriate services.” Meanwhile, a global interdisciplinary and cross-regional collaborative network should be established, with data sharing, technological empowerment and immigrant community participation at its core. Ultimately, this will promote the closed-loop transformation from research evidence to policy action, and realize the global equity vision of “legal status as a health insurance policy.”

## 4. Discussion

### 4.1. Overview of the study and trends in migrant health equity research

In this study, we conduct a comprehensive bibliometric analysis of migration health equity. A total of 409 scientific publications were retrieved from Scopus, and the development of health equity of migration over the past 20 years was analyzed. This study systematically summarizes the development context and trends of migration health equity, and serves as a basic reference and directional guide for future research in this field. Our research has uncovered the most productive countries, institutions, journals, and authors. It also identified the different stages of research trends in migration health equity, the most important research themes of migration health equity, and the evolution of the migration health equity.

In the past 20 years, the health equity of immigrants has attracted more attention, especially after 2013, and the average number of articles published has increased from 6.25 per year to 29.67 per year. From the analysis of the research categories, research on immigrant health equity has clear interdisciplinary characteristics. Examples include medicine, social sciences, the humanities and arts, nursing, environmental sciences, and psychology. However, an analysis of the double-map overlays of journals suggests that there may be fewer links between disciplines, such as economics. The study of health equity of immigrants from an economic perspective deserves further investigation.

### 4.2. Country, institution, and journal contributions to migrant health equity research

The US has the largest number of publications, especially the University of California, San Francisco, and the US has the largest scope and intensity of cooperation. American scholars have had strong cooperative relationships with many countries. This may be due to the advantages of the US as the world’s largest recipient of immigrants and a high proportion of scientific research investment,^[29]^ forming a virtuous cycle of “data-fund-output.” However, this has also led to research topics being biased towards the North American context, neglecting internal immigration issues in emerging market countries. Its high-intensity cooperation with countries like the UK and Canada (as shown in Fig. 4) has created an “English academic circle barrier.” Eighty-three percent of highly cited literatures are published in English journals, making research findings from non-English-speaking countries difficult to enter the core discourse system due to language and channel constraints, thus exacerbating the imbalance in global research contributions. The frequent adjustments to US state-level immigration policies have spurred policy evaluation research. In contrast, emerging market countries such as China, which are in a “development-first” stage, have placed immigrant health research at a lower priority in their national scientific research agenda. Ultimately, this has formed a global research pattern characterized by “Northern Hemisphere dominance and Southern Hemisphere marginalization.” Another reason may be that the US, as a developed market economy, has a large gap between the rich and poor, especially among different races, and there is a relatively obvious health gap problem.^[30]^ For example, the phenomenon of the “Latino health paradox” is that Latino immigrants face fewer fatal health problems than non-Latino whites, but this advantage does not extend to their children.^[31]^

Among emerging markets, China is also prominent in the network of cooperation. It has not only produced more publications, but has also conducted more extensive and intensive cooperation with the US. Moreover, emerging market countries pay less attention to migrant health equity than developed countries. This may be because emerging market countries are in the middle stage of industrialization and thus need to allocate most of their public budgets to infrastructure development and poverty reduction.^[32,33]^ In contrast, migrant health equity is likely to occupy a relatively low place on the agenda in these countries.^[34]^ This leads to a tilt in resource allocation toward “survival-oriented health needs.” Second, there is relatively little research and practical experience in the field of migrant health equity in emerging markets. Due to differences in historical, cultural, and social backgrounds, these countries may lack adequate experience and expertise in dealing with migrant health issues.^[35]^

It is precisely the formation of such a dominant position that has exerted certain negative impacts on global health equity research. Firstly, the research paradigms of high-income countries are defaulted to universal frameworks, while the intertwined challenges of “right to survival and right to health” faced by immigrants in low-income countries are overlooked. This bias may lead to global health policies being “incompatible with local contexts.” Despite the controversies over issue bias and uneven resource allocation, its positive impacts in terms of standardizing research methods, generating policy evidence, and promoting global agendas have provided developing countries with replicable paths and cooperation interfaces. given the complex and long-term nature of the migration process, emerging market countries should strengthen international cooperation with developed countries to ensure that migrants have the necessary health protection and equitable treatment before and after the migration process.^[36,37]^

The Social Science and Medicine journals hold a prominent position in migrant health equity research. Not only is the number of articles dominant, but also among the eleven highly cited papers, 3 of which are from the journal, which deeply discusses the multiple dimensions of migrant health equity. These highly cited articles delve into the multifaceted dimensions of migrant health equity, encompassing the intricate interplay of factors such as gender, social class, and place of origin, along with the specific impacts of national- and state-level immigration policies on health disparities experienced by Hispanic immigrants in the United Statse.^[26,28,38]^ This underscores not only the significant contribution of Social Science and Medicine to advance research on migrant health equity but also highlights the current research emphasis in this field, which centers on in-depth sociological analysis.

The formation of dominant positions among countries, institutions, and journals is closely tied to systemic factors such as funding allocation, geopolitical influences, and academic traditions. In the US, most immigration health funding is directed toward “disease prevention-medical services,” resulting in medical literature accounting for 75.31% of studies. This funding “Matthew Effect” skews research toward “quantifiable interventions” while neglecting qualitative analyses of structural discrimination. Geopolitical factors also shape research directions, as seen in Figure 4, where certain regions exhibit stronger collaborations while others show limited cooperation, possibly due to policy sensitivities. Additionally, path dependency in academic traditions plays a significant role. Europe’s “social medicine” tradition leads to 56 occurrences of “ social determinants of health” as a keyword, whereas the US’s biomedical paradigm yields 216 mentions of “medical services,” highlighting how different academic systems differentially shape research agendas.

Despite such biases stemming from dominant positions, as the issue of immigrant health equity garners increasing global attention, efforts to address this phenomenon are underway worldwide. The WHO has set a goal of eliminating medical discrimination against immigrants by 2030, yet our research reveals a significant gap between policy commitments and actual implementation. For instance, in the US, due to policy restrictions, the Medicaid coverage rate among Latino immigrants remains 34% lower than that of native residents^[26]^ (a phenomenon that corroborates the “disconnect between policy and practice” highlighted in previous studies). Such gaps underscore the urgency of bridging the divide between ambitious global health goals and on-the-ground realities. The United Nations Sustainable Development Goals (SDGs) emphasize promoting Universal Health Coverage through multisectoral collaboration to achieve health equity. However, our research analysis shows that only 23.72% of the literature incorporates a social science perspective, indicating that despite long-standing academic calls for interdisciplinary integration, relevant research remains insufficient.

Furthermore, this study also challenges certain assumptions in the global health agenda. The SDGs aim to reduce inequalities within and between countries, but the observed phenomenon of “health disadvantages among high-income Asian groups” in the research contradicts the inherent perception that “economic growth automatically improves health outcomes.” This aligns with the emphasis of the SDGs on “inclusive development” and also calls for further exploration of the nonlinear relationship between income growth of immigrant groups and health equity.

### 4.3. Key research topics and directions in migrant health equity

Several of the most popular research topics and directions related to health equity among immigrants were presented through keyword co-occurrence networks and highly cited articles. According to the co-occurrence of keywords and highly cited articles, current research directions can be summarized into the following categories, which can also be used as areas for future research:

1. Service delivery and supply side factors are crucial for migrant health equity. Providers such as governments, doctors, and community health workers impact immigrants’ healthcare access, quality, and trust, determining equity.^[39–42]^ Although Europe and other places have achieved the actual availability of health services, refugees, and migrants often have difficulty accessing health services due to economic and legal barriers, which reflects the problems of inadequate inclusion of health systems, information asymmetry, and lack of culturally sensitive care.^[39]^ Governments and public institutions must strengthen their structural readiness to provide culturally sensitive care. At the same time, the structure and interests of the physician team also have an important impact on immigrants’ health equity. For example, the reduction of pediatric and family doctors in the US and low interest in the health of immigrants make it more difficult for immigrant children to obtain medical services.^[40]^ In addition, access to supportive programs such as Medicaid, state-funded programs, and value-based care, which are critical to immigrants’ health, remains inaccessible because of their status.^[41]^ In conclusion, to achieve health equity for immigrants, the government, public institutions, health systems, and medical community must work together. Future research should pay more attention to the specific impacts of these factors and explore effective policies and measures to eliminate service delivery and supply-side barriers and promote health equity among migrants.
2. The effects of economic factors and social status on immigrants’ health equity In Asian countries such as India and China and high medical costs still hinder migrants’ access to healthcare despite better economic and health services provided by cities.^[43,44]^ This phenomenon reveals the important role of economic factors in the health equity of migrants and reflects that social status improvement is not sufficient to eliminate health inequalities. The US has shown significant racial, economic, and health inequalities amid the COVID-19 pandemic: counties with higher incomes, higher educational levels, and more diverse population structures have a higher risk of COVID-19 infection; in contrast, counties with higher poverty and disability rates have higher mortality rates. Meanwhile, African Americans have a much higher infection rate than whites and other ethnic groups. These findings further confirm the importance of social status in public health emergencies.^[45]^ These findings align with the framework proposed by Azizan et al, which identifies “socioeconomic stratification” as a core barrier to sports participation (a parallel to the dual impacts of economic disadvantage and social marginalization on immigrants’ access to health).^[46]^ Therefore, to improve the health status of migrants, the government and all sectors of society need to work together to reduce the health disparities between urban and rural areas and ethnic groups by making more equitable and reasonable medical resource allocation policies, improving medical security levels, and promoting economic development.
3. Demographic factors of health equity in migrants and migration issues. Existing studies have focused on demographic factors, such as age,^[47]^ gender,^[48,49]^ and race.^[50,51]^ For example, in terms of age, current research focuses more on the psychological and educational status of migrant children and emphasizes that promoting the health and well-being of these children is essential to prevent, treat, or cure health problems affecting the entire migrant family.^[52,53]^ However, it is important to note that despite the remarkable progress of these studies, there is still a significant shortfall in the field of research involving children from immigrant families, especially when probed at the family level.^[53]^ In exploring migrant health equity, given that children often migrate with the whole family, their developmental issues are also a challenge for the whole family. Therefore, family-based migration may play a pivotal role in the future research. A comprehensive understanding of its impact on health equity will help accurately address the integration challenges of immigrant families. To further explore the health equity of migrants, it is necessary to move beyond demographic factors and incorporate more complex factors, such as family-based migration.

### 4.4. Future research trends and policy recommendations

In addition to focusing on the perspective of family migration, future research should also prioritize the following 4 directions: first, strengthen in-depth research on internal migrants in emerging market countries and establish a global research network to compare policy differences across North America, Europe, and Southeast Asia. Second, emphasize the integration of multidisciplinary perspectives, including medicine, social sciences, and economics. Joint research teams should be formed to explore the nonlinear relationship between household income and health equity, while drawing on narrative research methods to develop large-scale narrative databases. Third, construct dynamic evaluation models that incorporate variables such as policy discrimination to track policy impacts, with a focus on identifying implementation barriers at the grassroots level. Fourth, conduct mechanistic research on the paradox of “health disadvantages among high-income Asian groups.” Meanwhile, referencing the “Latino health paradox,” track intergenerational changes in health indicators among migrant populations to reveal the dynamic relationship between social integration and health equity.

In terms of policy recommendations, based on research findings, it is necessary to advance interdisciplinary and international cooperation on a dual track. First, establish a global migrant health research fund, with a scale referencing the 120 million euros of the European Union’s “Horizon 2020” program.^[54]^ The fund should prioritize supporting research teams in low- and middle-income countries and require projects to include interdisciplinary collaboration. Meanwhile, develop an “Immigrant Health Research Toolkit” based on Scopus data to provide standardized research resources for regions with limited resources. Second, promote partnerships between research institutions in developed countries such as the US and Canada and universities in emerging market countries like China and India. Additionally, require high-income countries to open their original datasets simultaneously when publishing research and provide relevant training. Furthermore, under the framework of the Global Compact for Safe, Orderly and Regular Migration, signatory countries should be required to incorporate migrant health into their national health security strategies and establish special policies to help migrants meet their health needs. Finally, Policies in response to major public health emergencies should focus on the health needs of vulnerable groups and ensure the fair distribution of medical resources, build a global migrant health monitoring network based on big data to track indicators such as policy coverage and service utilization^[24]^ in 63 countries in real time, which will facilitate timely adjustments to migrant health policies.

### 4.5. Limitations

Based on a comprehensive analysis of bibliometrics and visualization techniques, this study sheds light on the current research landscape and the main dynamics of the academic frontier in the field of migrant health equity, providing valuable insights. It should be noted that the data in this study were derived from the Scopus database; therefore, the results of studies outside the Scopus database were not included. In addition, the VOSviewer tool is limited to abstracts and metadata at the time of analysis, and may not fully capture some study details. Simultaneously, given the temporal constraints associated with the publication cycle of academic journals, some high qualified research working newly published with lower citations or those are forthcoming may not be included in the analysis. Furthermore, continuous database updates may lead to subtle discrepancies between the search results and final number of publications included in the analysis. Nevertheless, we believe that this bibliometric report accurately reflects the overall development context and mainstream trends in migrant health equity.

## Acknowledgments

We thank the National Natural Science Foundation of China and Paperpal (https://preflight.paperpal.com/cn/partner/wolterskluwer/medicine) for the English language editing.

## Author contributions

**Data curation:** Jiaxin Liu, Jiahui Tian.

**Funding acquisition**: Rui Min.

**Project administration:** Rui Min, Fen Zhang.

**Resources:** Fen Zhang.

**Software:** Jiaxin Liu, Jiahui Tian.

**Validation:** Jiaxin Liu, Jie Xiao.

**Visualization:** Jiahui Tian, Jie Xiao.

**Writing – original draft:** Rui Min, Jiaxin Liu.

**Writing – review & editing:** Rui Min, Jiahui Tian.
